# Prognostic Value of Molecular Genetic Measurable Residual Disease (MRD) Monitoring in Pediatric Acute Myeloid Leukemia Expressing *KMT2A::MLLT10*

**DOI:** 10.1111/ejh.70019

**Published:** 2025-08-13

**Authors:** Emma Steidel, Eser Orhan, Mareike Rasche, Martina Pigazzi, Claudia Tregnago, Lina Marie Hoffmeister, Christiane Walter, Michael Dworzak, Nora Mühlegger, Nils von Neuhoff, Franco Locatelli, Dirk Reinhardt, Markus Schneider

**Affiliations:** ^1^ Department of Pediatric Hematology‐Oncology, Pediatrics III University Hospital of Essen Essen Germany; ^2^ Pediatric Research Network Essen Germany; ^3^ Division of Pediatric Hematology, Oncology and Stem Cell Transplant, Department of Women's and Children's Health University of Padua Padua Italy; ^4^ Department of Pediatrics, St Anna Children's Hospital Medical University of Vienna Vienna Austria; ^5^ St Anna Children's Cancer Research Institute Vienna Austria; ^6^ Department of Pediatric Hematology Oncology, IRCCS Ospedale Pediatrico Bambino Gesù University of Pavia Rome Italy; ^7^ German Society of Pediatric Oncology and Hematology (GPOH gGmbH) Essen Germany

**Keywords:** acute myeloid leukemia, childhood leukemia, complete remission, measurable (minimal) residual disease, real‐time polymerase chain reaction, risk stratification

## Abstract

**Objectives:**

Pediatric AML with *KMT2A::MLLT10* accounts for 10%–15% of *KMT2A*‐rearranged AML and is associated with poor prognosis. Lately, the assessment of measurable residual disease (MRD) by reverse transcription quantitative polymerase chain reaction (RT‐qPCR) has become an important tool for disease management; however, in the pediatric setting, it lacks standardized protocols. Therefore, we investigated the prognostic relevance of MRD monitoring by RT‐qPCR during high‐dose polychemotherapy in pediatric patients with AML expressing *KMT2A::MLLT10*.

**Methods:**

Using RNA sequencing, we determined the fusion breakpoints and designed RT‐qPCR assays for MRD monitoring. Bone marrow samples collected from 41 patients, who were treated in the AML‐BFM or AIEOP study, were analyzed for MRD by RT‐qPCR.

**Results:**

MRD positivity after the second treatment course resulted in a significantly worse probability of overall survival (pOS) compared to MRD negative patients (33.3% ± 19.2% vs. 80.6% ± 7.8%, *p* = 0.032). Moreover, the probability of event‐free survival (pEFS) (16.7% ± 15.2% vs. 76.9% ± 8.3%, *p* = 0.003) and cumulative incidence of relapse (CIR) (83.3% ± 40.8% vs. 19.2% ± 40.2%, *p* = 0.001) were significantly worse for patients in complete morphologic remission who remained MRD positive after the second treatment course.

**Conclusion:**

Thus, MRD monitoring enables the identification of a subgroup of pediatric patients with AML carrying *KMT2A::MLLT10* in complete morphologic remission with a dismal prognosis despite the current intensive therapy regimen.

**Trial Registration:**

AML‐BFM study 2004: ClinicalTrials.gov Identifier: NCT00111345; AML‐BFM registry 2012 and AML‐BFM study 2012: EudraCT 2013‐000018‐39; AML‐BFM registry 2017: DRKS number: DRKS00013030

## 1 Introduction

The prognosis for pediatric patients with acute myeloid leukemia (AML) has improved significantly in recent decades and currently reaches overall survival (OS) rates up to 76% [1, 2, 3, 4, 5, 6, 7]. Despite improvements in OS—largely attributed to intensive first‐line and relapse therapy as well as effective supportive care—event‐free survival (EFS) ranges from 46% to 59% [1, 2, 3, 4, 5, 6, 7]. High relapse rates of 25%–35% emphasize the need for more accurate risk stratification and risk‐adapted therapy [8, 9, 10, 11, 12, 13].

Several prognostic factors contribute to the clinical outcome of pediatric AML, in particular genetic abnormalities. Nowadays, the stratification to risk groups is mainly based on cytogenetic and molecular genetic characteristics and entails risk‐adapted treatment [14]. Beyond that, early response to initial treatment is an independent predictor of relapse risk and outcome, but morphologic assessment of response lacks sensitivity and specificity [15, 16, 17]. Remission rates based on morphologic techniques are > 85%, with a significant proportion of patients still having persistent occult disease [18, 19].

Measurable residual disease (MRD), previously denoted as minimal residual disease, describes the presence of remaining leukemic cells below the morphological limit of detection and is associated with increased risk of relapse and shorter survival [15, 17, 18, 20, 21].

Various methods are used for assessment of MRD: immunophenotyping by flow cytometry detects patterns of aberrant cell surface antigen expression on leukemic cells [22, 23, 24]. MRD monitoring using molecular genetic methods, such as reverse transcription quantitative polymerase chain reaction (RT‐qPCR) detects blast‐specific aberrations, for example fusion transcripts, which can be detected in approximately 40%–75% of all patients with AML [12, 25, 26, 27]. RT‐qPCR is a reproducible method with high sensitivity and specificity; it is therefore well suited as a tool for frequent MRD monitoring.

However, the assessment of MRD by RT‐qPCR in the pediatric setting lacks standardized protocols, cut‐off levels, and time points; therefore, limiting its widespread use in childhood AML.

Rearrangements of the *lysine methyltransferase 2a (KMT2A)* gene are particularly common in pediatric AML and occur in 20%–25% of newly diagnosed patients [28]. *KMT2A* is located on 11q23 and encodes a histone H3 lysine 4 methyltransferase. Rearrangements affecting *KMT2A* usually result in the formation of fusion proteins that deregulate cells at the transcriptional level, thus contributing to leukemogenesis [29]. To date, more than 100 fusion partners have been identified, which have a critical impact on gene expression profiles and clinical outcome [30, 31, 32].

AML with t(10;11) (p12;q23), leading to the expression of *KMT2A::MLLT10*, occurs in 2%–3% of pediatric patients with AML, predominantly in FAB M4 and M5 [12, 29, 30]. International study groups classify this subtype as a high‐risk acute leukemia with poor prognosis due to high risk of leukocytosis‐related complications and relapse [29, 30, 33, 34, 35]. Some studies revealed that MRD positivity is particularly prevalent in such high‐risk cohorts with an increased risk of relapse and death, indicating that these patients might benefit from intensified or targeted therapy depending on MRD monitoring [17, 18].

In this retrospective study, we characterize the role of molecular genetic MRD monitoring in pediatric patients with t(10;11) (p12;q23) expressing *KMT2A::MLLT10* for the assessment of initial treatment response, pEFS, and pOS.

## 2 Materials and Methods

### 2.1 Patients' Cohort

We investigated patients from 0 to < 18 years of age with a diagnosis of de novo AML between 01/2004 and 07/2020 treated in Germany or Austria and enrolled in the multicenter trials or registries of the AML‐BFM study group.

Patients with *de novo* AML, positivity for t(10;11) (p12;q23) *KMT2A::MLLT10*, a suitable bone marrow sample at the time of diagnosis, and at least one sample after induction therapy were eligible for study participation.

We excluded patients with secondary leukemia, patients with Down syndrome, myeloid leukemia (ML‐DS) or acute promyelocytic leukemia (APL) from analysis due to their unique biology and treatment.

A total of 40 patients registered in the AML‐BFM study showed positivity for t(10;11) preidentified by Fluorescence in situ hybridization (FISH) and HemaVision analyses (HemaVision‐28N, DNA Diagnostic, Risskov, Denmark). These patients were rescreened by an RNA‐based panel sequencing to detect fusion transcripts. This analysis confirmed the expression of *KMT2A::MLLT10* in 37 patients. In the remaining three patients, either no fusion transcript was detected or *PICALM::MLLT10* and *KMT2A::MLLT1* were detected, respectively.

In addition, the sequencing approach allowed the identification of 15 different fusion breakpoints of *KMT2A::MLLT10* in our analyzed patients.

Additionally, we included 19 patients with *KMT2A*::*MLLT10* from the Italian study group AIEOP.

**FIGURE 1:**
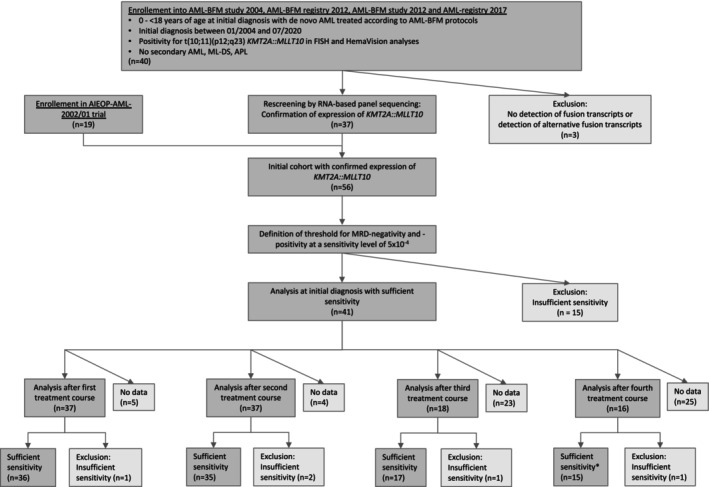
Consort diagram. Consort flow diagram showing patients of the AML‐BFM studies and registries from 2004 to 2017 and AIEOP‐AML 2002/01 study that have been included or excluded from the retrospective analysis. *No statistical analysis performed due to low patient numbers. AML, acute myeloid leukemia; APL, acute promyelocytic leukemia; BFM, Berlin‐Frankfurt‐Münster; FISH, fluorescence in situ hybridization; ML‐DS, Down syndrome myeloid leukemia; MRD, (minimal) measurable residual disease; *n*, number.

Between the different patients but also between samples of the same patients but of different time points, the sensitivity of MRD assays varied immensely. Thus, the sensitivity ranged from 8.8 × 10^−3^ to 1.7 × 10^−5^. To account for the big variation regarding sensitivity, we defined a threshold for MRD positivity and negativity at a sensitivity level of 5 × 10^−4^ for the evaluation. This resulted in the exclusion of individual time points of a few patient samples due to lack of sensitivity. The consort diagram displaying the composition of our study cohort is shown in Figure 1. MRD measurements were performed at diagnosis and after all four courses of high‐dose polychemotherapy.

Clinical and molecular details of the patients enrolled in the studies are shown in Table 1.

**TABLE 1:** Clinical characteristics of pediatric patients with AML expressing *KMT2A*::*MLLT10.*

			All patients	
			*n*	%
Total number of patients			41	
Age at diagnosis [years]	Median (range)		2.6 (0.1–17)	
	Categories	0–2 years	21	51.2
		3–10 years	12	29.3
		11–18 years	8	19.5
Sex	Female		18	43.9
	Male		23	56.1
FAB classification	M0		1	2.4
	M1/M2		3	7.3
	M3		0	0
	M4/M5		37	90.2
	M4eo		0	0
	M6		0	0
	M7		0	0
WBC count at diagnosis [× 10^9^/L]	Median (range)		19.5 (0.9–134.0)	
	WBC ≤ 100		35	85.4
	WBC > 100		6	14.6
	No data		0	0
Bone marrow blasts [%]	Blasts at diagnosis	Median (range)	80 (4–93)	
	Blasts at day 28	Median (range)	0 (0–65)	
	Blasts at day 56	Median (range)	0 (0–5)	
Remission status	CR		38	92.7
	NR		1	2.4
	No data		2	4.9
Relapse	Relapse		16	39.0
	No relapse		25	61.0
	Early relapse (< 1 year after diagnosis)		12	29.3
	Late relapse (> 1 year after diagnosis)		4	9.7
CR after relapse	Yes		8	19.5
	No		6	14.6
Interval between diagnosis and relapse [months]	Median (range)		9.1 (3.6–53.6)	
Secondary malignancy			2	4.9
HSCT	In total		38	92.7
	At first CR		35	85.4
	At nonresponse		1	2.4
	No HSCT at initial disease		2	4.9
Disease status at HSCT	CR		35	85.4
	NR		1	2.4
	NEL		0	0
	CR after relapse		2	4.9
	NR after relapse		0	0
Status	Dead		14	34.1
	Alive		27	65.9
Follow up [years]	Follow up in years		2.3 (0.2–10.2)	

Abbreviations: AML, acute myeloid leukemia; CR, complete remission; FAB classification, French‐American‐British classification; HSCT, hematopoietic stem cell transplantation; *n*, number; NEL, no evidence of leukemia; NR, non response; WBC, white blood cell.

National ethics committees and institutional review boards approved this study; patients or guardians provided written informed consent. The study was performed in accordance with the Declaration of Helsinki.

### 2.2 Treatment

AML‐BFM patients were enrolled in four different studies and registries. Patients in AML‐BFM study 2004 received either cytarabine, etoposide, and idarubicin or liposomal daunorubicin (AIE/ADxE) as the first induction course. The second induction course consisted of cytarabine and idarubicin (AI) or high‐dose cytarabine with mitoxantrone (HAM). Patients enrolled in AML‐BFM study 2012 received either cytarabine, liposomal daunorubicin, and etoposide or clofarabine (ADxE/CDxA) or AIE as the first induction. HAM was applied as the second induction course. Patients enrolled in AML‐registry 2012 and 2017 were treated with AIE and HAM as the induction regimen. Consolidation therapy (third and fourth course of treatment) consisted of AI with 2‐Chloro‐2‐desoxyadenosin (2‐CDA) and medium high‐dose cytarabine with mitoxantrone (haM) in AML‐BFM study 2004. Since AML‐BFM study and registry 2012, consolidation therapy for all high‐risk patients included AI and hAM.

AIEOP patients were enrolled in the AIEOP‐AML‐2002/01 trial, receiving two courses of induction chemotherapy, including idarubicin, cytarabine, and etoposide (ICE) [3].

Induction and consolidation therapy was followed by allogeneic hematopoietic stem cell transplantation (HSCT) in first complete remission.

MRD status by RT‐qPCR did not guide risk stratification or treatment intensity in any of the protocols.

### 2.3 Methods

#### 2.3.1 RNA Isolation

RNA was isolated using the RNeasy Mini Kit from Qiagen (Hilden, Germany) according to manufacturer's instructions.

#### 2.3.2 Next Generation Sequencing/RNA Sequencing

*KMT2A::MLLT10* breakpoint sequences were identified using the TruSight RNA Fusion Panel (Illumina, San Diego, CA, USA) according to the manufacturer's recommendations. Sequencing was performed on an Illumina MiSeqDX sequencer in research mode with 76 base pair (bp) paired‐end reads using the MiSeq Reagent Kit v3 (150‐cycle). For data analysis, including fusion calling, the RNA Fusion Analysis Module v2.0 of the local run manager and the Manta algorithm of the MiSeqDX system were used [36]. SnapGene software (www.snapgene.com; GSL Biotech LLC, San Diego, CA, USA) was used for breakpoint identification.

#### 2.3.3 qPCR Assay Design and Validation

Breakpoint determination revealed 15 different fusion breakpoints for our *KMT2A::MLLT10* positive cohort. Using PrimerBlast, we designed 15 different RT‐qPCR assays that first underwent initial testing including the patient sample as a positive control, a wildtype cDNA of healthy individuals as a specificity control, and a non‐template control. Using a dilution series of the patient cDNA, we determined the RT‐qPCR efficiency for each assay, making sure that it was between 80% and 105%. Further details regarding the RT‐qPCR assays are listed in Table S1.

#### 2.3.4 cDNA Synthesis

cDNA synthesis was performed using the SuperScript VILO cDNA Synthesis Kit (ThermoFisher Scientific).

#### 2.3.5 RT‐qPCR

The RT‐qPCR was carried out in a total volume of 25 μL containing 12.5 μL TaqMan Universal PCR Master Mix, 5 μL RNA‐free H_2_O, 2.5 μL Primermix (containing 3 μM each of forward and reverse primer and 2 μM of probe) and 5 μL of the respective cDNA.

Each patient sample was measured in triplicates with the appropriate assay and controlled with *ABL1* and *B2M* assays. Non‐target controls (water control), normal pool controls/wild‐type samples from healthy individuals, and *KMT2A::MLLT10* positive controls were also used in each RT‐qPCR run. The reaction conditions were 2 min at 50°C, 10 min at 95°C, followed by 50 cycles of 15 s at 95°C (denaturation) and 1 min at 60°C (annealing and extension). All reactions were performed with the Step One Software (Version 2.3).

PCR positivity was defined as amplification in at least 2 of 3 replicates with Ct values ≤ 40 (Cycling threshold of 0.09).

Using the relative quantification (ΔΔC_t_) method, RT‐qPCR MRD quantification was performed, where concordant efficiencies were regularly validated through routine assay testing. Sensitivities and MRD levels were calculated using the formulas proposed by Beillard et al. (2003), where the diagnostic MRD marker level is set to 1, and all subsequent measurements are expressed relative to the initial MRD level [37].

#### 2.3.6 Flow Cytometry

Flow cytometric immunophenotyping was performed according to the AIEOP‐BFM consensus guidelines and European Leukemia Network guidelines with adaptations for pediatric AML as published by Maurer‐Granofszky et al. [38, 39].

### 2.4 Definitions

The remission criteria were defined according to the Cancer and Leukemia Group B criteria at the end of intensification. Complete remission (CR) was defined as < 5% blasts in the bone marrow with sufficient bone marrow regeneration (neutrophile count > 1000/μL and platelets ≥ 100 000/μL) [40, 41].

Probability of overall survival (pOS) was defined as the time from diagnosis to death or last follow‐up. Probability of event‐free survival (pEFS) was calculated as the time from diagnosis to the first event (relapse, death of any cause, failure to achieve remission, or secondary malignancy) or last follow‐up. Failure to achieve remission was considered an event on day 0. Death within 42 days was considered early death (ED).

Cumulative incidence of relapse (CIR) was defined as the number of patients with relapse out of the total number of patients. Person‐time incidence rate (PIR) was defined as the number of relapses divided by the combined person‐time at risk.

### 2.5 Statistical Analyses

Statistical analyses were performed with IBM SPSS Statistics, version 27.

The Kaplan–Meier method was applied to estimate probabilities of survival. The log‐rank test was obtained to compare survival differences.

The cumulative incidence function was used to show the probability of CIR. The Kaplan–Meier estimator was used to consider censored data in the case of lost‐to‐follow‐up (LFU). The difference between groups was estimated by *t*‐test for paired samples.

All incidence functions and survival estimates are calculated as 5‐year‐point. All *p* values are descriptive and explorative. Differences with a *p* value < 0.05 were considered statistically significant. For the generation of diagrams, GraphPad Prism, version 10.2.0, was used.

## 3 Results

### 3.1 Patient Characteristics

We investigated 41 pediatric patients with AML expressing *KMT2A::MLLT10*. The median age at diagnosis was 2.6 years (range 0.1–17.0). In total, 56.1% (*n* = 23) of the patients were male. 92.7% (*n* = 38) of patients achieved complete remission at the end of intensification.

As pediatric AML with *KMT2A::MLLT10* is treated according to the high‐risk protocol, allogeneic HSCT was performed in 92.7% (*n* = 38) of patients, the majority in first complete remission (85.4%, *n* = 35). Three patients did not undergo HSCT (7.3%). One patient died early during polychemotherapy; two patients suffered an early relapse with subsequent progress and died before HSCT could be performed.

Sixteen patients (39.0%) experienced relapse at a median time of 9.1 months from diagnosis (range 3.6–53.6). Twelve patients (29.3%) experienced an early relapse within 1 year from diagnosis. Four patients (9.7%) relapsed more than 1 year after diagnosis.

Thirteen patients (31.7%) relapsed after first HSCT at a median time of 6.1 months (187 days). Median follow‐up was 2.3 years (range 0.2–10.2). Detailed patients' characteristics are presented in Table 1.

### 3.2 Worse Outcome of Patients With Positive MRD

Five‐year pOS of the entire cohort was 65.3% ± 7.5% (*n* = 41), while five‐year pEFS reached 55.3% ± 7.9%. CIR was 39.0% ± 49.4%.

In addition to the survival analysis of the entire cohort, we performed a second analysis including only patients who met the morphologic remission criteria; patients with morphologic blast persistence are known to have a worse prognosis. In the following, we will present the survival data of both analyses side by side.

**FIGURE 2:**
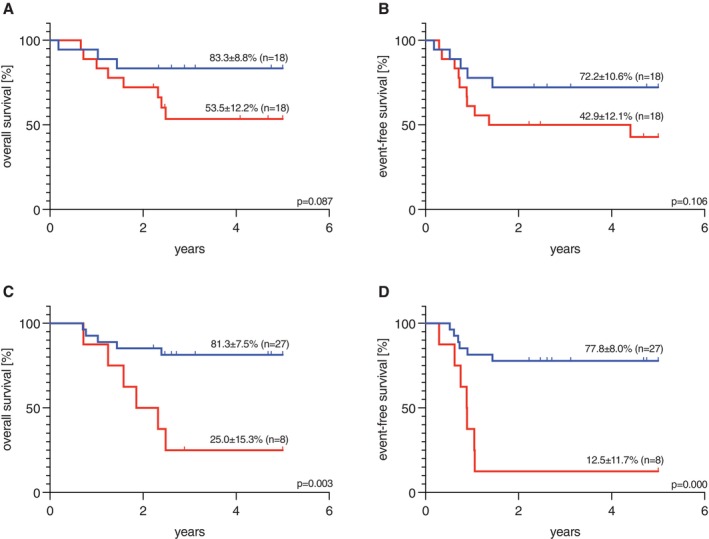
Survival data of pediatric patients with AML expressing *KMT2A::MLLT10* depending on MRD status during induction treatment. (A) 5‐year overall survival (pOS) of all patients with *KMT2A::MLLT10* with MRD negativity (83.3% ± 8.8%, *n* = 18) and MRD positivity (53.5% ± 12.2%, *n* = 18) after first treatment course (*p* = 0.087); (B) 5‐year event‐free survival (pEFS) of all patients with *KMT2A::MLLT10* with MRD negativity (72.2% ± 10.6%, *n* = 18) and MRD positivity (42.9% ± 12.1%, *n* = 18) after first treatment course (*p* = 0.106); (C) 5‐year pOS of all patients with *KMT2A::MLLT10* with MRD negativity (81.3% ± 7.5%, *n* = 27) and MRD positivity (25.0% ± 15.3%, *n* = 8) after second treatment course (*p* = 0.003); (D) 5‐year pEFS of all patients with *KMT2A::MLLT10* with MRD negativity (77.8% ± 8.0%, *n* = 27) and MRD positivity (12.5% ± 11.7%, *n* = 8) after second treatment course (*p* < 0.001); Significance was calculated with log‐rank test. MRD negative, MRD positive. MRD, (minimal) measurable residual disease; *p*, *p*‐value.

After the first treatment course, pOS in the entire cohort for MRD negative (*n* = 18) and MRD positive (*n* = 18) cases was 83.3% ± 8.8% and 53.5% ± 12.2%, respectively. However, this result did not reach statistical significance (*p* = 0.087, Figure 2A). The pEFS after the first course of therapy was 72.2% ± 10.6% in MRD negative patients vs. 42.9% ± 12.1% in MRD positive patients (*p* = 0.106, Figure 2B). With regard to CIR, results showed a trend toward worse CIR associated with MRD positivity after the first induction course: in MRD positive patients, CIR was 55.6% ± 51.1%, while patients with negative MRD showed a CIR of 16.7% ± 38.3% (*p* = 0.014).

**FIGURE 3:**
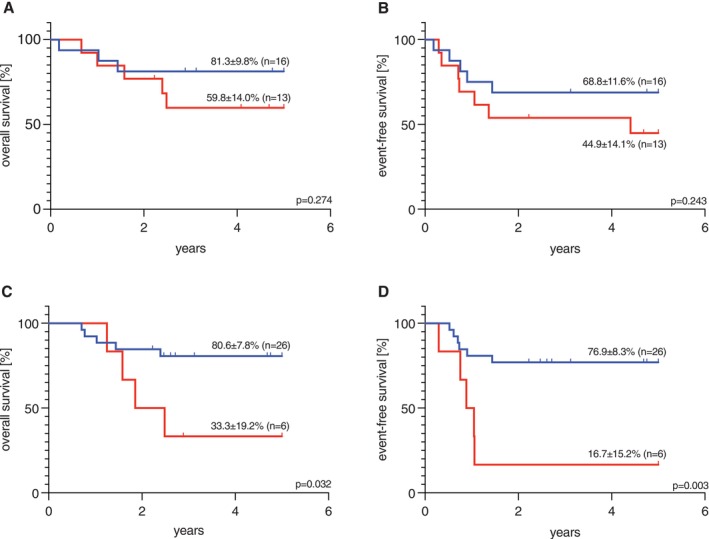
Survival data of pediatric patients with AML expressing *KMT2A::MLLT10* depending on MRD status considering patients without morphological evidence of blasts during induction treatment. (A) 5‐year overall survival (pOS) of patients with *KMT2A::MLLT10* and without morphological blast detection with MRD negativity (81.3% ± 9.8%, *n* = 16) and MRD positivity (59.8% ± 14.0%, *n* = 13) after first treatment course (*p* = 0.274); (B) 5‐year event‐free survival (pEFS) of patients with *KMT2A::MLLT10* and without morphological blast detection with MRD negativity (68.8% ± 11.6%, *n* = 16) and MRD positivity (44.9% ± 14.1%, *n* = 13) after first treatment course (*p* = 0.243); (C) 5‐year pOS of patients with *KMT2A::MLLT10* and without morphological blast detection with MRD negativity (80.6% ± 7.8%, *n* = 26) and MRD positivity (33.3% ± 19.2%, *n* = 6) after second treatment course (*p* = 0.032); (D) 5‐year pEFS of patients with *KMT2A::MLLT10* and without morphological blast detection with MRD negativity (76.9% ± 8.3%, *n* = 26) and MRD positivity (16.7% ± 15.2%, *n* = 6) after second treatment course (*p* = 0.003); Significance was calculated with the log‐rank test. MRD negative, MRD positive. MRD, (minimal) measurable residual disease; *p*, *p*‐value.

Considering patients in morphologic remission after the first therapy course, 29 patients with evaluable data were analyzed. In total, 16 cases were MRD negative, with a pOS of 81.3% ± 9.8%, while MRD positive patients (*n* = 13) showed a pOS of 59.8% ± 14% (*p* = 0.274, Figure 3A). The pEFS was 68.8% ± 11.6% vs. 44.9% ± 14.1% for MRD negative and MRD positive cases, respectively (*p* = 0.243, Figure 3B). The analysis of CIR after the first induction course confirmed the trend towards a worse outcome for MRD positive patients: CIR was 53.9% ± 51.9% (*n* = 13) compared to 18.8% ± 40.3% for MRD negative patients (*n* = 16). However, this analysis was not significant (*p* = 0.050).

The MRD analyses after the second therapy course showed a clear difference between MRD positive and MRD negative cases. After applying our sensitivity cut‐offs, 35 patients of the total cohort were evaluated after second induction: Here, 27 children were MRD negative and achieved a pOS of 81.3% ± 7.5%. Eight patients remained MRD positive with a pOS of 25% ± 15.3% (*p* = 0.003, Figure 2C). The pEFS in MRD negative patients was 77.8% ± 8% while it was only 12.5% ± 11.7% in MRD positive patients (*p* < 0.001, Figure 2D). CIR after the second course differed significantly depending on MRD results: Patients with MRD negativity showed a CIR of 18.5% ± 39.6% while the CIR for patients with MRD positivity was 87.5% ± 35.4% (*p* < 0.001). Survival data for this group are shown in Table 2.

**TABLE 2:** Outcome of pediatric patients with AML expressing *KMT2A*::*MLLT10* depending on MRD status.

	MRD status	After first treatment course		After second treatment course	
pOS	MRD negative	83.3% ± 8.8% (*n* = 18)	*p* = 0.087	81.3% ± 7.5% (*n* = 27)	*p* = 0.003
	MRD positive	53.5% ± 12.2% (*n* = 18)		25.0% ± 15.3% (*n* = 8)	
pEFS	MRD negative	72.2% ± 10.6% (*n* = 18)	*p* = 0.106	77.8% ± 8.0% (*n* = 27)	*p* < 0.001
	MRD positive	42.9% ± 12.1% (*n* = 18)		12.5% ± 11.7% (*n* = 8)	
CIR	MRD negative	16.7% ± 38.3% (*n* = 18)	*p* = 0.014	18.5% ± 39.6% (*n* = 27)	*p* < 0.001
	MRD positive	55.6% ± 51.1% (*n* = 18)		87.5% ± 35.4% (*n* = 8)	

Abbreviations: AML, acute myeloid leukemia; CIR, cumulative incidence of relapse; MRD, (minimal) measurable residual disease; *n*, number; *p*, *p*‐value; pEFS, probability of event‐free survival; pOS, probability of overall survival.

After the second course of therapy, MRD data from 32 patients in morphologic remission were evaluable: 26 patients were MRD negative and achieved a pOS of 80.6% ± 7.8%, whereas six children were MRD positive and had a pOS of 33.3% ± 19.2% (*p* = 0.032, Figure 3C). The pEFS between MRD negative and positive cases was also significantly different (*p* = 0.003), with values of 76.9% ± 8.3% and 16.7% ± 15.2%, respectively (Figure 3D). In the CIR analysis after second induction, the worse outcome for MRD positive patients in morphologic remission is underlined, with MRD negative patients having a CIR of 19.2% ± 40.2% and MRD positive cases showing a considerably higher CIR of 83.3% ± 40.8%. This analysis reached statistical significance (*p* = 0.001). Survival data for patients in morphologic remission are shown in Table 3.

**TABLE 3:** Outcome of pediatric patients with AML expressing *KMT2A*::*MLLT10* depending on MRD status considering patients in morphologic remission.

	MRD status	After first treatment course		After second treatment course	
pOS	MRD negative	81.3% ± 9.8% (*n* = 16)	*p* = 0.274	80.6% ± 7.8% (*n* = 26)	*p* = 0.032
	MRD positive	59.8% ± 14.0% (*n* = 13)		33.3% ± 19.2% (*n* = 6)	
pEFS	MRD negative	68.8% ± 11.6% (*n* = 16)	*p* = 0.243	76.9% ± 8.3% (*n* = 26)	*p* = 0.003
	MRD positive	44.9% ± 14.1% (*n* = 13)		16.7% ± 15.2% (*n* = 6)	
CIR	MRD negative	18.8% ± 40.3% (*n* = 16)	*p* = 0.05	19.2% ± 40.2% (*n* = 26)	*p* = 0.001
	MRD positive	53.9% ± 51.9% (*n* = 13)		83.3% ± 40.8% (*n* = 6)	

Abbreviations: AML, acute myeloid leukemia; CIR, cumulative incidence of relapse; MRD, (minimal) measurable residual disease; *n*, number; *p*, *p*‐value; pEFS, probability of event‐free survival; pOS, probability of overall survival.

After the third treatment course, the first course of consolidation, data from 17 patients were evaluable, hampering proper analysis of survival data. In total, 14 patients were MRD negative and three patients remained MRD positive. Of the 14 patients with MRD negativity, three patients relapsed and four patients died, whereas all three patients with MRD positivity suffered a relapse after 7, 10, and 16 months, respectively, and two of them died. Sample sizes after the fourth treatment course were too small to allow proper analysis (*n* = 15).

The PIR‐analysis for relapse did not reach statistical significance but showed a trend for higher PIR in patients with MRD positivity, in accordance with our results regarding the CIR. In patients with MRD negativity after the first course, the PIR was 0.02/year, while it was 0.13/year for patients with MRD positivity at the same time point (*p* = 0.99). After the second course of treatment, PIR for MRD negative patients remained at 0.03/year and increased to 0.32/year for MRD positive patients (*p* = 0.99). Thus, the PIR increased continuously with persistent MRD positivity until the time point after the second treatment course (data shown in Tables S2 and S3).

In summary, at all time points during high‐dose polychemotherapy, a positive MRD indicated a trend towards worse outcome in pOS as well as pEFS, with analyses after second induction showing a significant difference between MRD positive and MRD negative cases despite small patient numbers.

### 3.3 Comparison of Flow Cytometry and qPCR‐Data

Since flow cytometric MRD assessment is an established tool in most pediatric AML protocols, we compared the available data for discrepancies in results. MRD data from both methods were available for only 14 of 41 patients after first and second induction, hampering a comprehensive statistical analysis. For 9 additional patients, MRD data from both methods were provided for at least one common time point in therapy (after first or second induction). In 20 of these 23 cases, the flow cytometric and molecular genetic MRD diagnostics showed consistent results regarding MRD negativity and positivity. Discrepant results were found in three patients: two of the patients were MRD negative according to flow cytometric diagnostics after the first treatment course, while qPCR showed MRD positivity in both patients. MRD was consistently negative after the second treatment course using both methods. One patient showed MRD positivity in flow cytometric diagnostics, while qPCR indicated MRD negativity after first induction. Data after the second treatment course were not available for this patient.

## 4 Discussion

In this retrospective analysis of 41 pediatric patients with AML expressing *KMT2A::MLLT10*, we showed a prognostic relevance of MRD monitoring during high‐dose polychemotherapy for the assessment of treatment response.

The general survival data of our *KMT2A::MLLT10* cohort are in accordance with previous publications regarding *KMT2A*‐rearranged AML [30, 34]. The pOS (65.3% ± 7.5%) of our cohort with *KMT2A::MLLT10* is lower than recently published studies of the total AML‐BFM group, which confirm the poor prognosis of this high‐risk group. The pEFS (55.3% ± 7.9%), on the other hand, is roughly comparable to the results of the overall AML‐BFM cohort, as published in the study by Rasche et al. [5]. The CIR of 39.0% ± 49.4% is considerably higher than the most recently reported results of the AML‐BFM group, which once again emphasizes the importance of optimizing therapy in order to reduce relapse rates in this cohort. Minor differences between the results of these analyses can be explained by the setting of our study, as only patients were included who completed the induction therapy and therefore provided MRD data at the defined time points. Patients suffering early death or other complications preventing the regular administration of chemotherapy have not been evaluated in our analysis.

We analyzed differences in survival between MRD positive and negative patients with *KMT2A*::*MLLT10* after the first, second, and third courses of polychemotherapy. In particular, MRD positivity after the second induction course resulted in significantly worse pOS, pEFS, and a higher risk of relapse for patients in complete morphologic remission. This trend was already apparent in MRD analyses after the first induction and is confirmed in the further course of high‐dose polychemotherapy. Moreover, our results showing the increasing CIR and PIR in the case of persistent MRD positivity despite morphologic remission are in accordance with the survival data and emphasize the prognostic relevance of MRD status.

Previous studies have already indicated the prognostic relevance of MRD in pediatric AML [16, 17, 18, 19, 20]. Especially, Pigazzi et al. showed a significantly worse outcome of patients with t(8;21), leading to the expression of *RUNX1::RUNX1T1* and positive MRD after second induction [42]. This has been implemented in the current AIEOP‐AML‐BFM 2020 study in which a less than two log reduction led to restratification of the respective patients from standard into intermediate risk group. For *KMT2A*‐rearranged AML, Van Weelderen et al. recently showed that MRD positivity in flow cytometry after second induction is an adverse prognostic factor, which we could confirm in our study by means of RT‐qPCR [31].

Thus, MRD monitoring during induction therapy allows early identification of patients in complete remission who are at high risk of relapse despite the current intensive therapy regimen with polychemotherapy and HSCT for high‐risk AML. This emphasizes the need for intensified research regarding experimental therapies based on the individual relapse risk. For patients with *KMT2A*‐rearrangements, menin inhibitors may become a new treatment option [43]. On the other hand, the identification of patients at low risk of relapse is equally important to prevent patients from receiving unnecessary intensive treatment.

In a collaborative retrospective I‐BFM‐AML‐Study, Benetton et al. revealed that higher MRD values measured by qPCR had an adverse effect on posttransplant survival independent of genetic lesions [21]. The trend towards poor survival and high risk of relapse in patients remaining MRD positive before HSCT was also observed in our small cohort: all three patients who were still MRD positive after the third course of therapy received HSCT; all of them relapsed. Of the 14 patients with MRD negativity after the third course, 13 were transplanted, and only two patients experienced a relapse. Such observations underline the need for further analysis of MRD burden before HSCT and its impact on posttransplant survival.

Some studies from recent years performed MRD analyses not considering morphologic remission status and have included patients with morphologic evidence of blasts in their analyses [18, 44, 45]. By excluding patients with morphologic blast persistence, we aimed to get a more differentiated statement regarding the prognostic relevance of MRD in our cohort, as patients with blast persistence are known to have a worse prognosis anyway [8, 46].

Looking at the MRD results after second induction, it is notable that the pOS of MRD negative patients is roughly comparable in both analyses (80.6% ± 7.8% and 81.3% ± 7.5%). The pOS of MRD positive patients is lower when all patients are considered compared to the analysis excluding patients with morphological blast detection (25.0% ± 15.3% vs. 33.3% ± 19.2%). This finding is also evident for pEFS (12.5% ± 11.7% vs. 16.7% ± 15.2%). Therefore, the poor prognosis of patients with morphologic blast detection should be considered in analyses that do not differentiate according to morphologic remission criteria, to avoid misinterpretation of survival data.

In past years, larger studies have performed MRD monitoring mainly by flow cytometry [17, 18, 47]. With particular attention to broad applicability, this method is certainly advantageous for large cohorts. However, in AML, a leukemia‐specific antigen is missing, and a high heterogeneity of the leukemia‐associated immunophenotypes is encountered. In addition, the antigen expression pattern of leukemic blasts between initial diagnosis and eventual relapse proved to be instable in many cases [19, 48] Thus, the development of an appropriate antigen combination for reliable differentiation between leukemic blasts and normal hematopoietic cells remains challenging [49].

Campana et al. stated that the advantage of monitoring MRD by means of PCR is the strong association between the molecular abnormality and the leukemic clone, irrespective of cellular changes caused by therapy [50].

A few studies observed the detection of certain fusion transcripts in patients in long‐term remission and the instability of some mutations between diagnosis and relapse, which may limit the power of molecular genetic blast detection [15, 16]. We did not observe these features in our cohort; yet, such aspects should be considered in future analyses and studies. Due to the small cohort and limited flow data, we were only able to provide a basic comparison of flow cytometry and qPCR data. In two patients, qPCR was able to detect MRD positivity after the first induction, while the flow cytometric examination showed a negative result, which underlines the potentially higher sensitivity of molecular genetic methods. Nevertheless, larger‐scale comparisons of both methods are needed in the future to fundamentally confirm this finding. In general, it would be useful to take advantage of the broad applicability of flow cytometry combined with the high sensitivity of PCR methods [39]. Comprehensive consideration of flow cytometric data and PCR data may further refine clinical practice regarding risk stratification and individual therapeutic strategies.

Moreover, the question arises of how to deal with different subtypes and associated risk groups. Loken et al. were able to uncover significant differences in survival depending on MRD status, especially for high‐risk groups, but not for low‐risk cohorts [17]. Similarly, we could demonstrate the prognostic relevance of MRD positivity within a small homogeneous high‐risk cohort. Thus, broad analyses are needed that examine the prognostic relevance of MRD monitoring by RT‐qPCR across risk groups.

Summarized, our retrospective analysis of 41 pediatric patients with AML expressing *KMT2A::MLLT10* confirms the results of previous studies regarding the prognostic relevance of MRD in a homogeneously treated high‐risk cohort and emphasizes the need for future studies of frequent and long‐term MRD monitoring, which might further refine risk stratification and treatment design to improve the prognosis of patients with high‐risk AML.

## Author Contributions

**Emma Steidel:** conceptualization, data curation, investigation, methodology, project administration, supervision, visualization, writing – original draft preparation, review and editing. **Eser Orhan:** formal analysis, writing – review and editing. **Mareike Rasche:** conceptualization, methodology, writing – review and editing. **Martina Pigazzi:** data curation, investigation, writing – review and editing. **Claudia Tregnago:** data curation, investigation, writing – review and editing. **Lina Marie Hoffmeister:** data curation, investigation, writing – review and editing. **Christiane Walter:** data curation, investigation, writing – review and editing. **Michael Dworzak:** data curation, writing – review and editing. **Nora Mühlegger:** data curation, writing – review and editing. **Nils von Neuhoff:** resources, writing – review and editing. **Franco Locatelli:** funding acquisition, supervision, writing – review and editing. **Dirk Reinhardt:** funding acquisition, methodology, resources, supervision, writing – review and editing. **Markus Schneider:** conceptualization, data curation, investigation, methodology, project administration, supervision, writing – original draft preparation, review and editing.

## Ethics Statement

National ethics committees and institutional review boards approved this study, and patients or guardians provided written informed consent. The study was performed in accordance with the Declaration of Helsinki.

## Conflicts of Interest

The authors declare no conflicts of interest.

## Supporting information


**Table S1:** RT‐qPCR assays for different *KMT2A*::*MLLT10* breakpoints and housekeeping genes.
**Table S2:** Person‐time incidence rate/year in pediatric patients with AML expressing *KMT2A::MLLT10* depending on MRD status.
**Table S3:** Person‐time incidence rate/year in pediatric patients with AML expressing *KMT2A::MLLT10* depending on MRD status considering patients in morphologic remission.

## Data Availability

The data that support the findings of this study are available from the corresponding author upon reasonable request.
