# Supplementary material for: Prognostic Value of Molecular Genetic Measurable Residual Disease (MRD) Monitoring in Pediatric Acute Myeloid Leukemia Expressing KMT2A::MLLT10
Source: Eur J Haematol. 2025 Aug 13;115(5):493–504. doi: 10.1111/ejh.70019 (PMC12505838; doi:10.1111/ejh.70019)
Supplement: Supplementary file 1 — Table S1: RT‐qPCR assays for different KMT2A::MLLT10 breakpoints and housekeeping genes. Table S2: Person‐time incidence rate/year in pediatric patients with AML expressing KMT2A::MLLT10 depending on MRD status. Table S3: Person‐time incidence rate/year in pediatric patients with AML expressing KMT2A::MLLT10 depending on MRD status considering patients in morphologic remission. [file EJH-115-493-s001.docx]

**TABLE S1** RT-qPCR assays for different *KMT2A*::*MLLT10* breakpoints and housekeeping genes.

|  | Fusion breakpoint | RT-qPCR-efficiency [%] |
| --- | --- | --- |
| 1 | KMT2A E08 -MLLT10 E04 | 96.3 |
| 2 | KMT2A E07 - MLLT10 E15 | 100 |
| 3 | KMT2A E09 - MLLT10 E10 | 98.8 |
| 4 | KMT2A E07 - MLLT10 E16 | 98.7 |
| 5 | KMT2A E08 - MLLT10 E16 | 103.9 |
| 6 | KMT2A E09 - MLLT10 E06 | 93.9 |
| 7 | KMT2A E09 - MLLT10 E09 | 97.9 |
| 8 | KMT2A E06 - MLLT10 E15 | 100.9 |
| 9 | KMT2A E07 - MLLT10 E10 | 99.6 |
| 10 | KMT2A E08 - MLLT10 E09 | 89.2 |
| 11 | KMT2A E09 - MLLT10 E16 | 102.0 |
| 12 | KMT2A E07 - MLLT10 E09 | 87.0 |
| 13 | KMT2A E10 - MLLT10 E10 | 99.6 |
| 14 | KMT2A E07 - MLLT10 E17 | 89.0 |
| 15 | KMT2A E08 - MLLT10 E10 | 90.0 |
| 16 | ABL1 (housekeeping gene) | 88.0 |
| 17 | B2M (housekeeping gene) | 92.0 |

Abbreviations: RT-qPCR, Real time-quantitative polymerase chain reaction

**TABLE S2** Person-time incidence rate/year in pediatric patients with AML expressing *KMT2A::MLLT10* depending on MRD status.

| **Timepoint** | **MRD status** | **Patients (n)** | **Relapses (n)** | **Combined person-time at risk (person-years)** | **Person-time incidence rate/year** | **P-value** |
| --- | --- | --- | --- | --- | --- | --- |
| After first treatment course | MRD negative | 18 | 3 | 109.28 | 0.02 | 0.99 |
|  | MRD positive | 18 | 10 | 77.78 | 0.13 |  |
| After second treatment course | MRD negative | 27 | 5 | 160.87 | 0.03 | 0.99 |
|  | MRD positive | 8 | 7 | 21.67 | 0.32 |  |

Abbreviations: AML, Acute myeloid leukemia; MRD, (minimal) measurable residual disease; n, number

**TABLE S3** Person-time incidence rate/year in pediatric patients with AML expressing *KMT2A::MLLT10* depending on MRD status considering patients in morphologic remission.

| **Timepoint** | **MRD status** | **Patients (n)** | **Relapses (n)** | **Combined person-time at risk (person-years)** | **Person-time incidence rate/year** | **P-value** |
| --- | --- | --- | --- | --- | --- | --- |
| After first treatment course | MRD negative | 16 | 3 | 104.33 | 0.03 | 0.97 |
|  | MRD positive | 13 | 7 | 63.66 | 0.11 |  |
| After second treatment course | MRD negative | 26 | 5 | 153.51 | 0.03 | 0.99 |
|  | MRD positive | 6 | 5 | 18.63 | 0.27 |  |

Abbreviations: AML, Acute myeloid leukemia; MRD, (minimal) measurable residual disease; n, number
